# Rosuvastatin Reduces Neuroinflammation in the Hemorrhagic Transformation After rt-PA Treatment in a Mouse Model of Experimental Stroke

**DOI:** 10.3389/fncel.2018.00225

**Published:** 2018-08-02

**Authors:** Dan Lu, Yanfang Liu, Hongcheng Mai, Jiankun Zang, Lingling Shen, Yusheng Zhang, Anding Xu

**Affiliations:** ^1^Department of Neurology and Stroke Center, The First Affiliated Hospital, Jinan University, Guangzhou, China; ^2^Clinical Neuroscience Institute of Jinan University, Guangzhou, China

**Keywords:** rosuvastatin, hemorrhagic transformation, microglia, astrocytes, blood-brain barrier, NF-κB, MAPK pathway

## Abstract

Hemorrhagic transformation (HT) is a serious complication that stimulates inflammation during reperfusion therapy after acute ischemic stroke. Rosuvastatin, a 3-hydroxymethyl-3-methylglutaryl coenzyme A (HMG-CoA) reductase inhibitor, might improve the outcome of HT by inhibiting neuroinflammation. This study aimed to explore the protective effects of rosuvastatin against HT after recombinant tissue plasminogen activator (rt-PA) treatment in mice with experimental stroke via the attenuation of inflammation. A total of one hundred sixty-nine male BALB/c mice were used in the experiment. HT was successfully established in 70 mice that were subjected to 3 h of middle cerebral artery occlusion (MCAO) followed by a 10 mg/kg rt-PA injection over 10 min and reperfusion for 24 h. The mice were then administered rosuvastatin (1 mg/kg, 5 mg/kg) or saline (vehicle). The brain water content and neurological deficits (wire hang and adhesive removal somatosensory tests) were assessed at 24 h after rt-PA reperfusion following MCAO surgery. The morphology, blood-brain barrier (BBB) permeability and number of astrocytes and microglia were assessed by immunohistochemistry, electron microscopy and western blotting at 24 h after rt-PA reperfusion following MCAO surgery. Rosuvastatin protected against impaired neurological function and reversed the BBB leakage observed in the HT group. The increased activation of astrocytes and microglia and secretion of inflammatory factors caused by HT damage were significantly attenuated by high-dose rosuvastatin treatment vs. normal-dose rosuvastatin treatment. Related inflammatory pathways, such as the nuclear factor kappa B (NF-κB) and mitogen-activated protein kinase (MAPK) pathways, were downregulated in the rosuvastatin-treated groups compared with the HT group. In conclusion, our results indicate that rosuvastatin is a promising therapeutic agent for HT after rt-PA reperfusion following MCAO surgery in mice, as it attenuates neuroinflammation. Additionally, high-dose rosuvastatin treatment could have a greater anti-inflammatory effect on HT than normal-dose rosuvastatin treatment.

## Introduction

Hemorrhagic transformation (HT) is a serious complication that occurs after acute ischemic stroke (Álvarez-Sabin et al., 2013; Wang et al., 2015). In clinical practice, after the administration of intravenous recombinant tissue plasminogen activator (IV-rt-PA) within the therapeutic window, the rate of type two parenchymal hemorrhage within 7 days is 6.8%, with a 90-day mortality rate as high as 17.9% (Emberson et al., 2014). Studies have shown that disturbances in the blood-brain barrier (BBB), which is composed mainly of endothelial cells, pericytes and astrocytes, occur throughout the process of HT (Kelly et al., 2006; Mishiro et al., 2012; Ozkul-Wermester et al., 2014). Emerging data have shown that the oxidative stress and overexpression and release of proinflammatory cytokines caused by rt-PA reperfusion are associated with BBB disruption (Wang et al., 2015). Furthermore, increasing studies in the literature have shown that inflammation related to the interaction between microglia and astrocytes but not astrocytes alone contributes to ischemia-induced HT and oedema (del Zoppo et al., 2012).

Statins, also known as 3-hydroxy-3-methylglutaryl-coenzyme A (HMG-CoA) reductase inhibitors, are well known for having beneficial effects on vascular events by lowering cholesterol levels (Postmus et al., 2014) and are used as neuroprotectants in experimental brain ischemia (Spence, 2014). *In vivo* studies have shown that statins induce time- and concentration-dependent reductions in Aβ production, and the reduced production of Aβ has been attributed to reductions in neuroinflammation (Hosaka et al., 2013). One study has suggested that reduced chronic neuroinflammation might be a key mechanism underlying statin-induced neuroprotection (McFarland et al., 2014). Furthermore, statins play a protective role against neurodegenerative conditions, including vascular dementia, Alzheimer’s disease (AD) and Parkinson’s disease (PD; Mandas et al., 2014). However, whether statins protect against HT and the related mechanisms in mice have not been determined.

Rats who were treated with rosuvastatin (a synthetically derived statin) immediately post-spinal cord injury demonstrated reduced inflammatory cell infiltration, tumor necrosis factor alpha (TNF-α) expression, myeloperoxidase activity, nitric oxide levels and caspase-3 activity in caudal spinal cord tissue (Kahveci et al., 2014). Pretreatment with rosuvastatin significantly reduced lipopolysaccharide (LPS)-induced interleukin 1 beta (IL-1β) and TNF-α release (Kahveci et al., 2014). Because the protective effect of rosuvastatin relies on the modulation of several signaling transduction pathways, including the nuclear factor kappa B (NF-κB), phosphatidylinositol 3-kinase and protein kinase B (PI3K/Akt) and c-Jun N-terminal kinase (JNK) pathways (Li et al., 2015; Liu et al., 2017), determining the potential role played by rosuvastatin in neuroinflammation-related diseases is critical.

In this study, experiments were performed to demonstrate our hypothesis that rosuvastatin protects against HT in middle cerebral artery occlusion (MCAO) mice by attenuating inflammation.

## Materials and Methods

### Animals

A total of 169 male BALB/c mice (10–12 weeks old, weighing 22–25 g) were used. All animal procedures were performed in strict accordance with the National Institutes of Health guidelines (NIH publication no. 8023, revised 1978). Experimental protocols were approved by the Competent Ethics Committees of Jinan University, and efforts were made to reduce the total number of animals used as well as their potential pain and suffering.

### Stroke Model, Experimental Design and Experimental Groups

**Figure 1:**
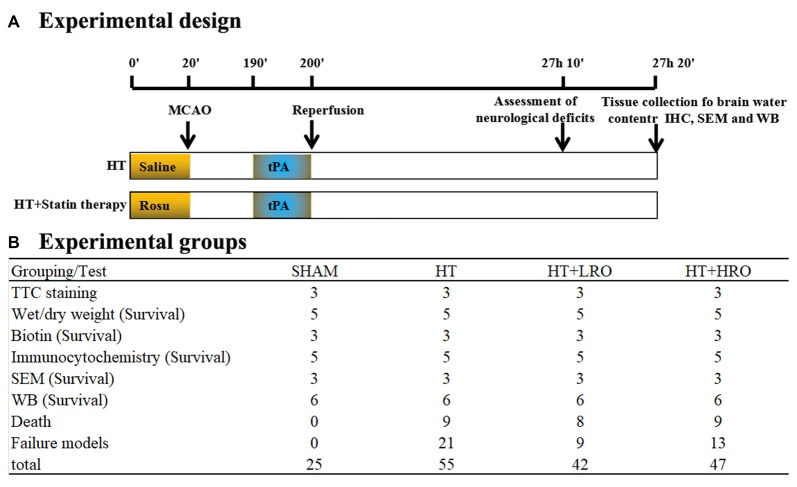
Schematic overview of the stroke model and group allocation. **(A)** Timeline depicting the study design. The locomotor activity and sensory assessments were performed at 23 h and 50 min after reperfusion and 10 min prior to tissue collection at 24 h after reperfusion. **(B)** Summary of the mice in the various groups. The total number of mice does not include those excluded due to death by anesthesia, poor recovery after surgery, or model failure.

One-hundred and forty-four mice were anesthetized with isoflurane in air (4% for inducing anesthesia, 1.5% for maintaining anesthesia; RWD Life Science, Shenzhen, China). The temperature of the mice was maintained at 37.0 ± 0.5°C using a heating pad, and focal ischemia was induced using an intraluminal filament (Mehra et al., 2012). A midline incision was made in the neck, and the left common carotid artery, external carotid artery and internal carotid artery were isolated. Briefly, the stump of the external carotid artery was cut, and a filament made of nylon string coated with silicon (MSMC23B104PK100, RWD Life Science, Shenzhen, China) was carefully inserted into the internal carotid artery and advanced 11 mm from the carotid artery bifurcation or until resistance was encountered. Changes in regional cerebral blood flow were monitored using a laser Doppler blood flow meter in the left MCA region (Mehra et al., 2012) to confirm successful MCAO. After 3 h of MCAO, thrombolysis was conducted via a tail vein injection of rt-PA (10 mg/kg, Actilyse, Boehringer Ingelheim Pharma GmbH and Co., UK) in saline for 10 min; then, the filament was carefully withdrawn to induce vascular recanalization/reperfusion for 24 h. The liquid was warmed to 37°C prior to intraperitoneal and tail vein injection. In addition, the mice remained on the heating pad in fresh air and were monitored (blood pressure, heart rate, and physical activity) until they showed movement. The operation time was kept to less than 10 min from the induction of anesthesia. During the 12 h of daytime, the mice were given 1 ml of jelly two times by oral feeding. Then, the mice were kept in a cage and allowed free access to jelly and water in a dish during the 12 h of night-time (Lourbopoulos et al., 2017). Mice in the sham-operated group served as controls and were treated with an equal volume of saline administered via the tail vein. At 24 h after reperfusion and the assessment of neurological deficits, the mice were deeply anesthetized with 5% isoflurane. After acute bleeding, tissue samples were collected for brain water content measurements and immunoblotting; other samples were collected after heart perfusion experiments for the assessment of BBB integrity and immunofluorescence, immunohistochemistry and electron microscopy (Figure 1A).

Mice in this experiment were divided randomly into four groups (Figure 1B): (1) SHAM: sham-operated mice treated with saline as a control; (2) HT: mice pretreated with saline for 10 min before 3 h of MCAO followed by tPA treatment and 24 h of reperfusion; (3) normal-dose rosuvastatin therapy for HT-LRO: mice pretreated with 1 mg/kg rosuvastatin for 10 min before 3 h of MCAO followed by tPA treatment and 24 h of reperfusion; and (4) high-dose rosuvastatin therapy for HT-HRO: mice pretreated with 5 mg/kg rosuvastatin for 10 min before 3 h of MCAO followed by rt-PA treatment and 24 h of reperfusion. Twenty-six mice died because of failure to tolerate anesthesia or severe postoperative complications, such as subarachnoid hemorrhage and forty-three mice were excluded due to model failure; these mice are excluded from the following tests (Figure 1B).

### Assessment of Neurological Deficits

Behavioral tests, including the wire hang and adhesive removal somatosensory tests, were conducted 24 h after reperfusion.

In the wire hang test (Wu et al., 2010), mice were placed midway on a wire (50 × 0.15 cm) mounted between two platforms 40 cm above the ground. Two days before formal experiments were conducted, all mice were familiarized with the technique of grasping the wire by the forelimbs. Their performance was observed for a maximum of 1 min, and exact scores were calculated. A mouse scored three points if it grasped the wire with both hind paws, two points if it grasped the wire with one hind paw, and one point if it did not grasp the wire with either hind paw. The suspension times were recorded and scored as follows: 0 points for 0–4 s; one point for 5–9 s; two points for 10–14 s; three points for 15–19 s; four points for 20–24 s; five points for 25–29 s; and six points ≥30 s. Each testing session consisted of three trials, and the interval between the trials was 5 min.

The adhesive removal somatosensory test is a sensitive method for assessing sensorimotor deficits (Modo et al., 2000; Bouet et al., 2009; Freret et al., 2009). All mice were familiarized with the testing environment before formal experiments were conducted. Two small, circular adhesive-backed paper patches (diameter: 10 mm) were used as bilateral tactile stimuli occupying the distal-radial region on the wrist of each forelimb. Each mouse was then returned to its respective home cage, and the time required to remove each stimulus was recorded (with a maximum limit of 120 s). Three trials were conducted per day, and individual trials were separated by at least 10 min.

### Brain Water Content Measurement

The brain water content was measured using the wet/dry method (Manaenko et al., 2011). Briefly, after mice from each group were decapitated under deep anesthesia with 5% isoflurane, the whole brains were immediately removed. Each brain was weighed (wet weight [WW]) on an electronic analytical balance (Sartorius, Gottingen, Germany) and then dried at 100°C for 24 h to determine the dry weight (DW). The brain water content (%) was calculated as ([WW − DW]/WW) × 100%.

### TTC Staining and Infarct Volume Measurement

At 24 h after reperfusion, following deep anesthesia with 5% isoflurane, four mice were selected randomly from each group and perfused transcardially with cold saline. The brains were quickly removed and sectioned coronally into 1-mm slices and used for 2,3,5-triphenyltetrazolium chloride (TTC) staining. A Canon camera (Guangzhou, Guangdong, China) was used to capture images of the slices before they were stained with 2% TTC and incubated in a dark chamber at 37°C for 20 min. The infarct volumes were calculated as follows: infarct volume (%) = (contralateral hemisphere volume—ipsilateral hemisphere + infarct volume)/contralateral hemisphere volume × 100%. The hemispheric volumes were measured using Image-Pro^®^ Plus (Version 6.0 for Windows™, National Institutes of Health, Bethesda, MD, USA; Zhu et al., 2016).

### HE Staining

Brain tissue was collected and fixed in 4% paraformaldehyde (PFA) for 12 h after the heart perfusion experiments. The brains were cut into halves and immersed in 30% sucrose in distilled water overnight at 4°C before being embedded in optimal cutting temperature (OCT) compound. Cryosections with a thickness of 10 μm were dewaxed, dehydrated and stained with hemeatoxylin and eosin (H&E) for morphological evaluation. Each section was examined under 400× magnification using a Leica microscope (Vizna, Germany). The area of red blood that overflowed from the BBB into the brain tissues was obtained as average values and is expressed as the ratio of the area of red blood to the area of the corresponding high-power (HP) field.

### Assessment of BBB Integrity

Mice were deeply anesthetized with 5% isoflurane. Fifty milliliters of 0.01 mol/l PBS containing EZ-link-sulfo-NHS-biotin (0.5 mg/ml; Thermo Fisher Scientific, Waltham, MA, USA) was used to perfuse the left ventricle of the heart over 5 min, followed by perfusion with 50 ml of ice-cold 1% PFA in 0.01 mol/l PBS. The brains were then dissected, fixed in 4% PFA for 1 h at room temperature, cut into halves and immersed in 30% sucrose in distilled water overnight at 4°C before being embedded in OCT compound. Cryosections with a thickness of 10 μm were blocked in 0.01 mol/l PBS + 10% normal goat serum + 0.3% Triton X-100 for 30 min at room temperature. The sections were then incubated with fluorescein isothiocyanate streptavidin (FITC-streptavidin; 1:200, Yeasen, Shanghai, China) for 1 h at room temperature; 4′,6-diamidino-2-phenylindole (DAPI; 1:250; Beyotime Biotechnology, Shanghai, China) was used to stain nuclei for 10 min before the slides were mounted. Images of the peri-infarct area, were captured using a confocal laser scanning microscope (Leica SP8, Vizna, Germany). The statistical data were generated from a total of three mice; these mice were used for three independent experiments, and three replicate slices were analyzed per group in each independent experiment.

### Immunofluorescence

To assess the number of activated astrocytes, we used a cryostat (Thermo Fisher Scientific, Waltham, MA, USA) to prepare coronal cryosections (6 μm). After being permeabilized with 0.3% Triton X-100 in PBS for 30 min, the sections were blocked with 5% goat serum for 1 h. Then, the sections were incubated at 4°C overnight with the primary monoclonal anti-glial fibrillary acidic protein antibody (anti-GFAP; 1:250, Santa Cruz Biotechnology). After being washed with 0.01 mol/l PBS for 5 min, the sections were incubated three times with horseradish peroxidase-conjugated secondary antibodies (1:250; Yeason, Shanghai, China) at room temperature for 1 h. After being washed with 0.01 mol/l PBS for 5 min three times, DAPI was used to stain nuclei for 10 min before the slides were mounted. The number of positive cells was counted in the peri-infarct cortex (200× magnification) under a fluorescence microscope (Leica, Vizna, Germany), and the number of cells was analyzed as positive cells/total cells using ImageJ 1.50 software (National Institutes of Health, Bethesda, MD, USA).

### Immunohistochemistry

To assess the number of activated microglia, we used a cryostat to obtain coronal cryosections (6 μm). The sections were permeabilized with 0.3% Triton X-100 in PBS for 30 min and then blocked with 5% goat serum for 1 h. The sections were then incubated at 4°C overnight with the primary anti-ionized calcium-binding adaptor molecule 1 antibody (Iba-1; 1:250, Santa Cruz Biotechnology, Dallas, TX, USA). The sections were incubated with horseradish peroxidase-conjugated secondary antibodies (1:250; Yeason, Shanghai, China) at room temperature for 1 h and then stained with 3,3-diaminobenzidin (Wanleibio, Shanghai, China) for 10 min. After the sections were washed three times with 0.01 mol/l PBS for 5 min, they were stained with hemeatoxylin for 10 min to label nuclei and were then dehydrated and mounted with neutral gum. The number of positive cells in the peri-infarct cortex was counted (200× magnification) under a fluorescence microscope (Leica, Vizna, Germany), and the number of cells was analyzed as positive cells/total cells using ImageJ 1.50 software (National Institutes of Health, Bethesda, MD, USA).

### Electron Microscopy

Mice were anesthetized with 5% isoflurane and transcardially perfused with a cold saline solution followed by 2.5% glutaraldehyde in 0.1 M phosphate buffer. Then, the brains were isolated and dissected. The brains were sectioned, dehydrated, embedded in epoxy resin and then visualized using a HITACHI transmission electron microscope (HITACHI, Tokyo, Japan) at 80 kV. The sections were selected as previously described, and five areas in the ipsilateral peri-infarct cortex in each section were chosen.

### Immunoblotting

At 24 h after recanalization, the cortex was obtained for immunoblotting as previously described. Proteins obtained from the peri-infarct cortex were subjected to sodium-dodecyl sulfate polyacrylamide gel electrophoresis and electrically transferred to a polyvinylidene difluoride membrane before being incubated with specific antibodies. Primary antibodies against the following mediators were used: TNF-α, cyclooxygenase 2 (Cox-2), inducible nitric oxide synthase (iNOS), IL-6, phospho-NF-κB p65 (p-p65), phospho-inhibitory subunit of NF-κB-α (p-IκBα), p-c-Jun, phosphorylated c-Jun-N-terminal kinase (p-JNK), phosphorylated mitogen-activated protein kinase (MAPK) p38 (p-p38), β-actin (1:1000; all from Cell Signaling Technology, Danvers, MA, USA) and IL-1β (1:1000; Wanleibio, Shanghai, China). The membranes were incubated for 1 h with the appropriate secondary antibody (anti-rabbit IgG, anti-mouse IgG; 1:5000; Yeasen). The antibodies were visualized by enhanced chemiluminescence (ECL Plus; Beyotime Biotechnology). ImageJ (NIH, Bethesda, MD, USA) was used to analyze the band intensity. For individual samples, each value was normalized to that of β-actin.

### Statistical Analyses

All data were analyzed using SPSS (Windows version 13.0; SPSS Inc., Chicago, IL, USA). Values are expressed as the mean ± SEM. Statistical differences among the groups were assessed by one-way ANOVA. All analyses were performed using GraphPad Prism six for Windows (GraphPad Software, Inc., La Jolla, CA, USA). In all tests, *P*-values of 0.05 or less were considered to indicate significance.

## Results

### Rosuvastatin Attenuated HT in Mice

**Figure 2:**
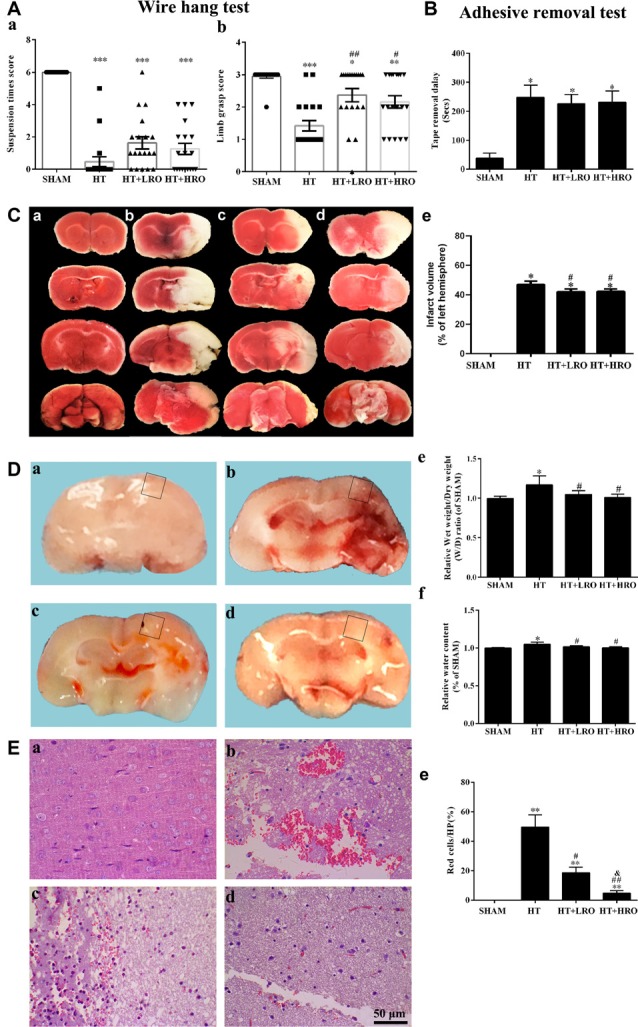
Rosuvastatin attenuated hemorrhagic transformation (HT) after recombinant tissue plasminogen activator (rt-PA) reperfusion in mice. **(A)** Quantitative analysis of the wire hang test (*n* = 5 in each group) in the SHAM (sham-operated), HT (3 h of middle cerebral artery occlusion (MCAO) followed by tPA reperfusion), HT+LRO (rosuvastatin pretreatment for 10 min before MCAO followed by tPA reperfusion), and HT+HRO (rosuvastatin 5 mg/kg pretreatment for 10 min before MCAO followed by tPA reperfusion) groups. **(a)** The hang duration scores were expressed as the mean ± SEM, and between-group differences were assessed by one-way ANOVA followed by Tamhane’s T2 test (homogeneity of variance was not determined), *n* = 5. ***P < 0.001 vs. the SHAM group. **(b)** The grasp power scores were expressed as the mean ± SEM, and between-group differences were assessed by one-way ANOVA followed by Tamhane’s T2 test (homogeneity of variance was not determined), *n* = 5. *P < 0.05, **P < 0.01, and ***P < 0.001 vs. the SHAM group, ^##^*P* < 0.01 vs. the HT group, ^#^*P* < 0.05 vs. the HT group. **(B)** Adhesive removal test (*n* = 5 in each group) in the SHAM, HT, HT+LRO, and HT+HRO groups. The adhesive removal duration scores are expressed as the mean ± SEM, and between-group differences were assessed by one-way ANOVA followed by Tamhane’s T2 test (homogeneity of variance was not determined), *n* = 5. *P < 0.05 vs. the SHAM group. **(C)** Representative images of TTC staining from the four groups (**a–d** for the SHAM, HT, HT+LRO and HT+HRO groups, respectively). **(e)** The infarction volume scores were expressed as the mean ± SEM, and between-group differences were assessed by one-way ANOVA followed by the LSD test (homogeneity of variance was determined), *n* = 3 per group. *P < 0.05 vs. the SHAM group, ^#^*P* < 0.05 vs. the HT group. **(D)** Representative images of gross brain sections from the four groups (**a–d** for the SHAM, HT, HT+LRO and HT+HRO groups, respectively). **(e)** Relative wet weight/dry weight (WW/DW) ratios in the four groups. **(f)** Brain water contents (%) in the four groups. The values were expressed as the mean ± SEM, the determination of which was followed by the LSD test (homogeneity of variance was determined), *n* = 5. *P < 0.05 vs. the SHAM group, ^#^*P* < 0.05 vs. the HT group. **(E)** The pathological changes in the number of red blood cells in the ischemia penumbra determined under high-power (HP) microscopy in the four groups (**a–d** for the SHAM, HT, HT+LRO and HT+HRO groups, respectively). **(e)** The values were expressed as the mean ± SEM, and between-group differences were assessed by one-way ANOVA followed by Tamhane’s T2 test (homogeneity of variance was not determined), *n* = 5. **P < 0.01 vs. the SHAM group, ^#^*P* < 0.05 and ^##^*P* < 0.01 vs. the HT group, ^&^*P* < 0.05 vs. the HT+LRO group.

To examine whether rosuvastatin influenced the severity of cerebral hemorrhage, we evaluated locomotor activity using the wire hang test and assessed sensation by performing the adhesive removal test. The wire hang test was used to assess the strength and stamina of the mice. Deficit scores were assessed using the max hang duration in the HT group (***P < 0.001 vs. the SHAM group). Neither normal- nor high-dose statin therapy significantly shortened the falling latency in the SHAM group compared to the HT group (Figure 2Aa). The adhesive removal test showed that mice in the HT group required more time than did those in the SHAM group (*P < 0.05), while there were no significant differences between the statin-therapy groups and the HT group (Figure 2B). The grasp power assessment showed that the HT mice treated with saline achieved an average limb grasp score of 1.42 ± 0.16 (***P < 0.001 vs. 2.95 ± 0.05 in the SHAM group), while those treated with normal-dose statin therapy showed an average grasp power score significantly higher than that of the HT mice (2.36 ± 0.21 vs. 1.42 ± 0.16, respectively, ^##^*P* < 0.01). The mice treated with high-dose statin therapy showed a significantly higher grasp power score than did the HT mice (2.16 ± 0.19 vs. 1.42 ± 0.16, respectively, ^#^*P* < 0.05; Figure 2Ab). Meanwhile, as shown in Figures 2Ca–e, more infarction was observed in the HT mice than in the SHAM mice (*P < 0.05), while less infarction was observed in the HT+LRO and HT+HRO groups than in the HT group (^#^*P* < 0.05). In the gross slices of the brain dissected after saline perfusion, we found that the amount of bleeding was increased in the HT group, and the statin therapy improved the bleeding in the HT+LRO and HT+HRO groups (Figures 2Da–d). Furthermore, to verify the phenomenon, we then evaluated the water content 24 h after ischemia, which revealed that rt-PA reperfusion injury increased the brain water content compared with the sham operation. However, this effect was lower in the rosuvastatin-treated groups (1 mg/kg, water content: 1.05 ± 0.05, ^#^*P* < 0.05; 5 mg/kg, water content: 1.00 ± 0.04, ^#^*P* < 0.05) than in the HT group (water content: 1.17 ± 0.11) (Figure 2De). Similarly, the brain water content (%) in the HT group was significantly higher than that in the SHAM group, while the percentage in the HT+HRO group was lower than that in the HT group (^#^*P* < 0.05; Figure 2Df). Experiments of greater precision were used to assess the bleeding by HE staining (Figures 2Ea–e). Using HP microscopy, we found that the blood leakage in the HT group was significantly higher than that in the SHAM group (red blood percentage: 49.55 ± 8.33%, **P < 0.01 vs. the SHAM group), while the blood leakage was significantly lower in the rosuvastatin-treated groups than in the HT group (1 mg/kg, red blood percentage: 18.59 ± 3.72%, ^#^*P* < 0.05; 5 mg/kg, water content: 3.82 ± 1.70, ^##^*P* < 0.05 vs. the HT group), especially in the high-dose rosuvastatin-treated group (^&^*P* < 0.05 vs. the HT+LRO group).

Thus, taken together, our results show that rosuvastatin substantially improved the HT-induced deficit in locomotor activity and attenuated the blood leakage resulting from HT-induced damage after rt-PA therapy reperfusion in mice. In addition, high-dose rosuvastatin treatment improved the attenuation of the HT-associated bleeding more than the normal-dose rosuvastatin treatment.

### Rosuvastatin Decreased BBB Leakage

**Figure 3:**
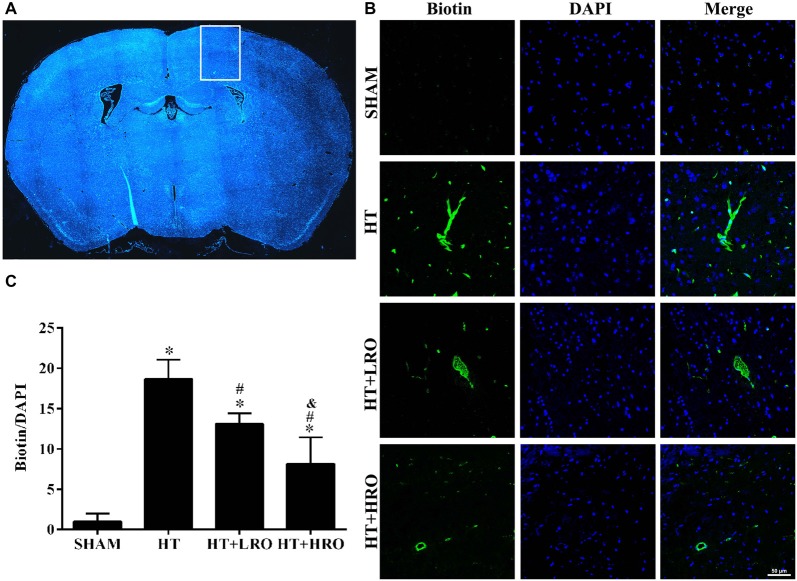
Rosuvastatin decreased blood-brain barrier (BBB) leakage. **(A)** In the peri-infarct area indicated by the white box, images were captured and tissue samples were collected, as shown in the HT group as an example. **(B)** Representative photomicrographs of sections stained with 4′,6-diamidino-2-phenylindole (DAPI) and biotin from the peri-infarct areas of the brain at 24 h after reperfusion. Scale bar = 50 μm. **(C)** Relative biotin leakage ratios were analyzed by the ratio of biotin- to DAPI-positive cells in each group (*n* = 3 in each group). Values were expressed as the mean ± SEM, the determination of which was followed by the LSD test (homogeneity of variance was determined). *P < 0.05 vs. the SHAM group, ^#^*P* < 0.05 vs. the HT group, ^&^*P* < 0.05 vs. the HT+LRO group.

Disturbances in the BBB have been implicated in the HT process (Khatri et al., 2012; Ozkul-Wermester et al., 2014). We next utilized EZ-link-sulfo-NHS-biotin to examine the levels of BBB permeability, and if the BBB permeability increased, biotin leakage was examined. Biotin leakage images were captured in the peri-infarct areas of the brain slides (Figure 3A). Biotin leakage in the HT group was significantly higher than that in the SHAM group; however, the BBB permeability was significantly attenuated in the rosuvastatin-treated groups, especially in the high-dose rosuvastatin-treated group (Figures 3B,C <0.05). These results indicate that rosuvastatin protects against HT by decreasing BBB leakage.

### Rosuvastatin Attenuated Inflammation Levels

**Figure 4:**
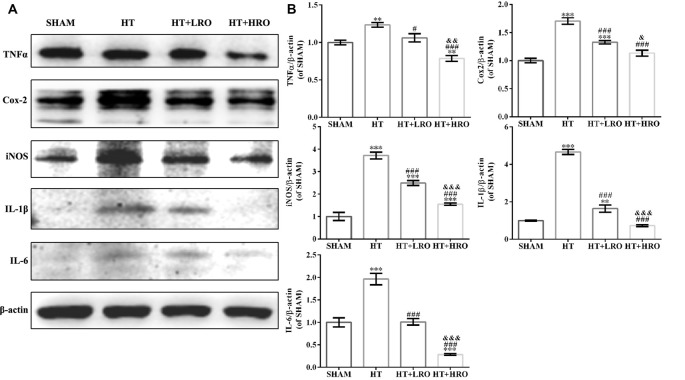
Rosuvastatin attenuated inflammation levels. **(A)** Immunoblotting analysis of tumor necrosis factor alpha (TNF-α), cyclooxygenase 2 (Cox-2), inducible nitric oxide synthase (iNOS), interleukin 1 beta (IL-1β) and IL-6 expression levels at 24 h after reperfusion in each group (*n* = 6 in each group). **(B)** The results are normalized to β-actin. Values are expressed as the mean ± SEM, the determination of which was followed by the LSD test (homogeneity of variance was determined). **P < 0.01 and ***P < 0.001 vs. the SHAM group, ^#^*P* < 0.05 and ^###^*P* < 0.001 vs. the HT group, ^&^*P* < 0.05, ^&&^*P* < 0.01 and ^&&&^*P* < 0.01 vs. the normal-dose rosuvastatin-treated group.

Reperfusion with rt-PA causes the release of proinflammatory cytokines that disturb the BBB permeability, and protective effects exerted by statins are associated with the reduced expression of neuroinflammatory mediators, such as TNF-α and IL-1β (McFarland et al., 2014, 2017; Wang et al., 2015). Thus, to further investigate whether rosuvastatin reduces HT via attenuating inflammation, we examined inflammation levels using western blotting. rt-PA reperfusion injury significantly increased the expression of the inflammatory cytokines TNF-α (**P < 0.01), Cox-2 (***P < 0.001), iNOS (***P < 0.001), IL-1β (***P < 0.001) and IL-6 (***P < 0.001) relative to their expression levels in the SHAM group. However, the levels of TNF-α (^#^*P* < 0.05), Cox-2 (^###^*P* < 0.001), iNOS (^###^*P* < 0.001), IL-1β (^###^*P* < 0.001) and IL-6 (^###^*P* < 0.001) were significantly lower in the rosuvastatin-treated groups than in the HT group, and levels of TNF-α (^&&^*P* < 0.01), iNOS (^&^*P* < 0.05), IL-1β (^&&&^*P* < 0.001) and IL-6 (^&&&^*P* < 0.001) were significantly lower in the high-dose rosuvastatin-treated group than in the normal-dose rosuvastatin-treated group (Figure 4). Therefore, these findings suggest that both a normal and a high dose of rosuvastatin exert neuroprotective effects by attenuating inflammation and that the high dose of rosuvastatin might reduce the release of proinflammatory factors more than the normal dose.

### Rosuvastatin Inhibited Astrocyte Activation

**Figure 5:**
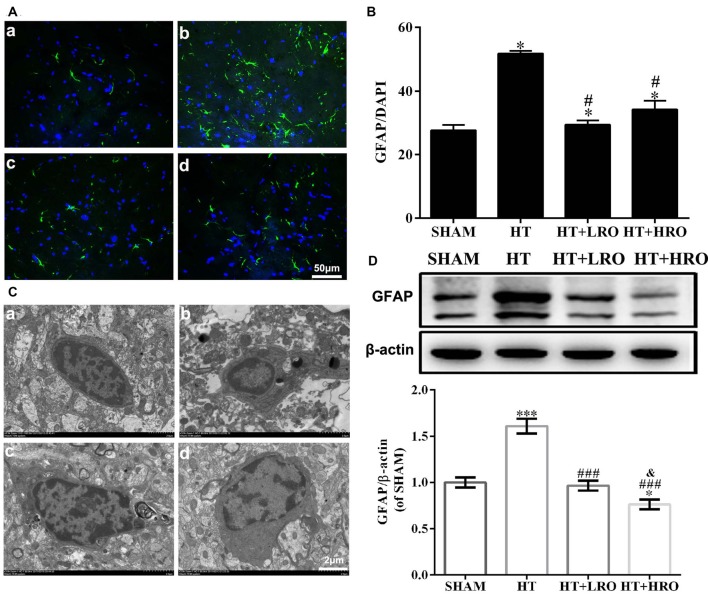
Rosuvastatin inhibited astrocyte activation. **(A)** Representative images of DAPI and glial fibrillary acidic protein (GFAP) staining, as described above. Scale bars: 50 μm. **(B)** The quantified GFAP immunofluorescence intensity at 24 h after reperfusion; the results were normalized to DAPI. **(C)** Transmission electron microscopy image showing the ultrastructure of astrocytes. Scale bar: 200 nm. **(D)** GFAP expression levels, as determined by western blotting (*n* = 6 in each group); the results were normalized to β-actin. The values were expressed as the mean ± SEM (**a–d** for the SHAM, HT, HT+LRO and HT+HRO groups, respectively, as shown in Figure 1). *P < 0.05 and ***P < 0.001 vs. the SHAM group,^#^*P* < 0.05 and ^###^*P* < 0.001 vs. the HT group, ^&^*P* < 0.05 vs. the normal-dose rosuvastatin-treated group.

Astrocytes participate in the formation of the BBB and act as major support cells in the central nervous system (CNS; Argaw et al., 2012; Colombo and Farina, 2016). The levels of GFAP, an astrocyte marker, in the HT group were significantly higher than those in the SHAM and rosuvastatin-treated groups (Figures 5Aa–d for the SHAM, HT, HT+LRO and HT+HRO groups, respectively, and Figure 5B, *P* < 0.05). The levels of GFAP in the rosuvastatin-treated groups were lower than those in the HT group (Figure 5B, *P* < 0.05). Ultrastructural analysis showed cell shrinkage, increased accumulation of abnormal electron-dense materials, nuclear condensation, and basement membrane thickening in the HT group. In comparison, less necrosis was observed in the rosuvastatin-treated groups (Figures 5Ca–d for the SHAM, HT, HT+LRO and HT+HRO groups, respectively). To further assess the number of astrocytes in peri-infarct areas at 24 h after reperfusion, GFAP expression levels were detected. Significantly inhibited astrocyte activation was observed in the rosuvastatin-treated groups compared with the HT group (Figure 5D, ^###^*P* < 0.001), whereas significantly inhibited astrocyte activation was observed in the high-dose rosuvastatin-treated group compared with the normal-dose rosuvastatin-treated group (Figure 5D, ^&^*P* < 0.05). Thus, these results indicate that rosuvastatin decreased BBB leakage via inhibiting astrocyte activation and that astrocyte activation was more inhibited by the high rosuvastatin dose than the normal rosuvastatin dose.

### Rosuvastatin Inhibited Microglial Activation

**Figure 6:**
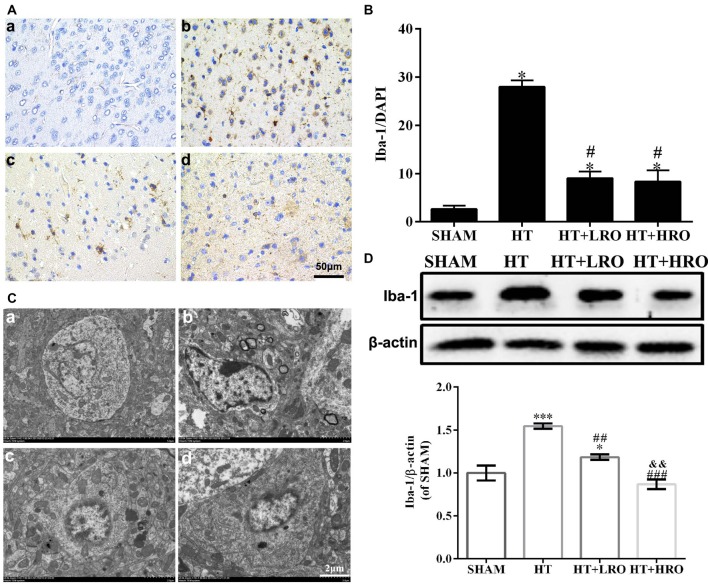
Rosuvastatin inhibited microglial activation. **(A)** Representative images of DAPI and Iba-1 staining, as described above. Scale bars: 50 μm. **(B)** The quantified Iba-1 immunofluorescence intensity at 24 h after reperfusion; the results were normalized to DAPI. **(C)** Transmission electron microscopy image showing the ultrastructure of microglia. Scale bar: 200 nm. **(D)** Iba-1 expression levels, as determined by western blotting (*n* = 6 in each group); the results are normalized to β-actin. The values were expressed as the mean ± SEM, the determination of which was followed by the LSD test (homogeneity of variance was determined; **a–d** for the SHAM, HT, HT+LRO and HT+HRO groups, respectively, as shown in Figure 1). *P < 0.05 and and ***P < 0.001 vs. the SHAM group, ^#^*P* < 0.05, ^##^*P* < 0.01 and ^###^*P* < 0.001 vs. the HT group, ^&&^*P* < 0.01 vs. the normal-dose rosuvastatin-treated group.

Previous studies have demonstrated that activated glia also play a vital role in CNS inflammation and behave as another major immune cell in the CNS; thus, we next examined microglial activation (Kettenmann et al., 2011; Xiao et al., 2013). Analysis of Iba-1, a microglial reactivity marker, was performed to verify microglial activation. The integrated density of Iba-1 staining was significantly higher in the HT group, while treatment with rosuvastatin significantly inhibited this increase (Figures 6Aa–d for the SHAM, HT, HT+LRO and HT+HRO groups, respectively, and Figure 6B <0.05). Ultrastructural analysis showed cell shrinkage, increased accumulation of abnormal electron-dense materials, and an increased nuclear-to-cytoplasmic ratio in the HT group compared with the rosuvastatin-treated groups, which showed less necrosis (Figures 6Ca–d for the SHAM, HT, HT+LRO and HT+HRO groups, respectively). To further assess the number of microglia in the peri-infarct areas at 24 h after reperfusion, Iba-1 expression levels were determined. Significantly inhibited microglial activation was observed in the rosuvastatin-treated groups relative to the HT group (Figure 6D, ^##^*P* < 0.01 and ^###^*P* < 0.001). Significantly inhibited microglia activation was also observed in the high-dose rosuvastatin-treated group relative to the normal-dose rosuvastatin-treated group (Figure 6D, ^&&^*P* < 0.01). Consistent with the above results, these findings indicate that rosuvastatin decreased BBB leakage via inhibiting microglial activation and that microglial activation was more inhibited in the high-dose rosuvastatin-treated group than in the normal-dose rosuvastatin-treated group.

### Rosuvastatin Protected Against HT via Inhibiting the NF-κB Pathway

**Figure 7:**
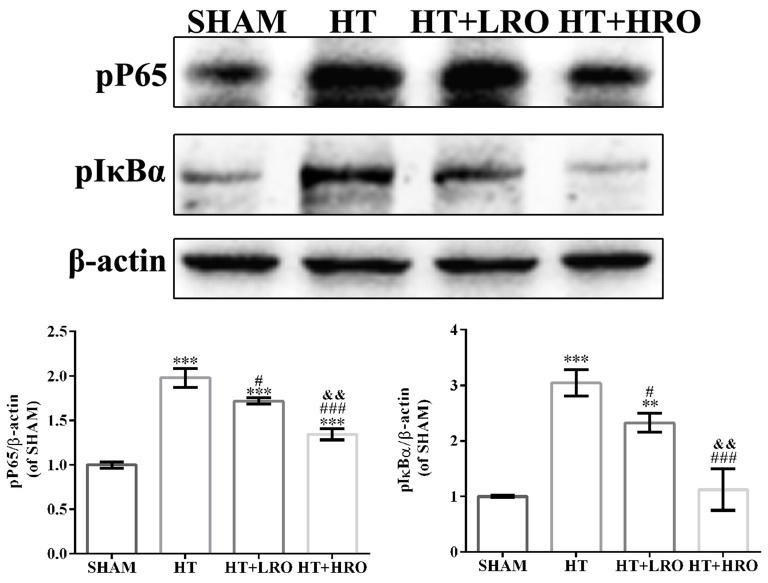
Rosuvastatin protected against HT via inhibiting the NF-κB pathway. Western blot analysis of phospho-NF-κB p65 (p-p65) and phospho-inhibitory subunit of NF-κBα (p-IκBα) expression levels in the NF-κB pathway at 24 h after reperfusion (*n* = 6 in each group); the results are normalized to β-actin. The values are expressed as the mean ± SEM.**P < 0.01 and ***P < 0.001 vs. the the SHAM group, ^#^*P* < 0.05 and ^###^*P* < 0.001 vs. the HT group, ^&&^*P* < 0.01 vs. the normal-dose rosuvastatin-treated group.

Considering the crucial influence of canonical NF-κB on glial cell activation and inflammatory responses following HT, we measured the expression levels of proteins in the NF-κB pathway (Hayden and Ghosh, 2008; Sun and Ley, 2008). Blocking IκBα phosphorylation potentially inhibits the activation of molecules in the NF-κB pathway, such as p-NF-κB p65, thus downregulating inflammatory factors, including TNF-α, Cox-2, iNOS, IL-1β and IL-6. Thus, we determined the protein levels of the key NF-κB pathway factors p65 and IκBα. The levels of p-p65 and p-IκBα were substantially greater in the HT group than in the SHAM group (Figure 7, ^#^*P* < 0.05), while these levels were significantly lower in the rosuvastatin-treated groups than in the HT group (Figure 7, **P < 0.01 and ***P < 0.01). The levels of p-p65 and p-IκBα were significantly lower in the high-dose rosuvastatin-treated group than in the normal-dose rosuvastatin-treated group (Figure 7, ^&&^*P* < 0.01). Thus, these results indicate that rosuvastatin attenuated HT by inhibiting the NF-κB pathway.

### Rosuvastatin Protected Against HT via Inhibiting the MAPK Pathway

**Figure 8:**
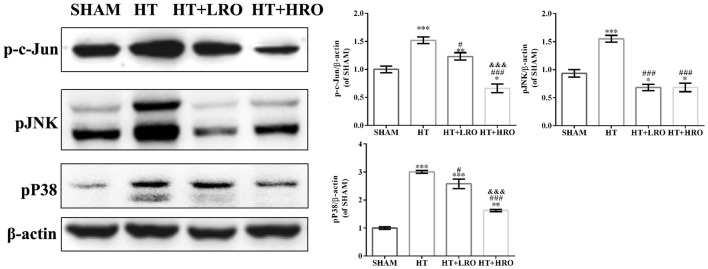
Rosuvastatin protected against HT via inhibiting the mitogen-activated protein kinase (MAPK) pathway. Western blot analysis of p-c-Jun, phosphorylated c-Jun-N-terminal kinase (p-JNK) and p-p38 expression levels at 24 h after reperfusion (*n* = 6 in each group); the results are normalized to β-actin. The values are expressed as the mean ± SEM. *P < 0.05, **P < 0.01 and ***P < 0.001 vs. the SHAM group, ^#^*P* < 0.05 and ^###^*P* < 0.001 vs. the HT group, ^&&&^*P* < 0.05 vs. the normal-dose rosuvastatin-treated group.

As a member of the MAPK family related to neuronal survival, JNK has been shown to increase stroke injury upon activation, and p38 signaling has been shown to exacerbate stroke-induced inflammatory responses (Barone et al., 2001; Kuan et al., 2003; Cui et al., 2007; Nithianandarajah-Jones et al., 2012). Thus, we next investigated the role of rosuvastatin in the MAPK pathway using western blotting. Activated MAPKs can stimulate IκB kinase, release NF-κB dimers from the inactive cytoplasmic NF-κB/IκB complex and induce the nuclear translocation of NF-κB. The levels of p-c-Jun, p-JNK and p-p38 were higher in the HT group than in the SHAM group (Figure 8, ***P < 0.05) and the rosuvastatin-treated groups (Figure 8, ^#^*P* < 0.05, ^###^*P* < 0.001). The expression levels of p-c-Jun and p-p38 in the high-dose rosuvastatin-treated group were lower than those in the normal-dose rosuvastatin-treated group (Figure 8, ^&&&^*P* < 0.001). Thus, taken together, these results suggest that the attenuation of the inflammatory response by rosuvastatin is involved in the MAPK pathway.

## Discussion

Inflammation is a major factor contributing to brain damage and nervous system dysfunction after intracerebral hemorrhage (ICH; Campos et al., 2013; Zhou et al., 2014). Rosuvastatin has been used as a cholesterol-lowering drug for a long time and is more effective than other statins (McTaggart et al., 2001; Gullestad et al., 2012). Rosuvastatin has been demonstrated to exert neuroprotective effects by promoting anti-inflammatory responses in rat animal models of brain ischemia, thus improving their outcome. However, whether rosuvastatin exerts neuroprotective effects against secondary damage, including hemorrhaging after thrombolysis, is not clear. The major findings of this study are that rosuvastatin, at both normal and high doses, contributes to preventing HT after intravenous rt-PA thrombolysis in mice with brain ischemia by activating glial cells.

In addition to blood leakage due to disruption of the BBB, which significantly upregulates the activation of microglia/macrophages (Khatri et al., 2012; Ozkul-Wermester et al., 2014) and astrocytes (del Zoppo et al., 2012), the elevated expression of the proinflammatory cytokines IL-1β and IL-6, the activation of their transcription factor NF-κB, and the expression of the inflammatory enzymes Cox-2 and iNOS are considered the chief mechanisms underlying HT. Studies have shown that rosuvastatin can alleviate motor dysfunction and neuropathic pain detected by the wire hang and adhesive removal somatosensory tests (Bouet et al., 2009). Meanwhile, TNF-α, Cox-2, iNOS, IL-1β and IL-6 levels are ameliorated by NF-κB inactivation, and the numbers of microglia and astrocytes are decreased in rosuvastatin-treated mice. As proinflammatory cytokines produced by microglia and astrocytes, TNF-α and IL-1β are closely related to oedema formation and BBB dysfunction, leading to further brain injury in HT, as determined by increased WW/DW ratios (Ma et al., 2017). However, decreased WW/DW ratios after rosuvastatin treatment in HT mice resulted from the inhibition of TNF-α, Cox-2, iNOS, IL-1β and IL-6. Furthermore, NF-κB transcription factors are present in the cytosol in an inactive state complexed with inhibitory IκB proteins, and activation occurs via the phosphorylation of IκBα at Ser32 and Ser36, followed by proteasome-mediated degradation that results in the release and nuclear translocation of active NF-κB in HT mice. However, rosuvastatin might prohibit the activation of IκBα and NF-κB, thus suppressing the subsequent release of inflammatory cytokines (Asahi et al., 2005; Cordle and Landreth, 2005; Barone et al., 2011). We believe that treatment with rosuvastatin early in thrombolysis therapy will reduce the release of inflammatory cytokines, thus prohibiting oedema formation and BBB dysfunction.

The MAPK family is a group of serine/threonine protein kinases comprising several members, including extracellular signal-regulated kinases 1/2, JNK and p38 (Nithianandarajah-Jones et al., 2012). The importance of MAPKs in stroke, especially JNK and p38, is well documented in the literature. More specifically, JNK activation has been shown to increase stroke injury via the enhancement of neuronal apoptosis, and the genetic and pharmacological inhibition of JNK both improve the outcome after stroke (Kuan et al., 2003; Cui et al., 2007). p38 signaling activation exacerbates stroke-induced inflammatory responses and leads to poorer outcomes (Barone et al., 2011). IL-1β and other inflammatory cytokines can increase the phosphorylation of specific amino acid sequences in these components and then activate MAPKs. Activated MAPKs can stimulate IκB kinase, release NF-κB dimers from the inactive cytoplasmic NF-κB/IκB complex and induce the nuclear translocation of NF-κB (Kim et al., 2005; Joo et al., 2007). The activation of p38 and JNK in microglia and astrocytes via various pathways has also been demonstrated to be essential for IL-1β, IL-6, TNF-α, Cox-2 and iNOS expression (Koistinaho and Koistinaho, 2002). Indeed, the pharmacological inhibition of p38 and JNK by rosuvastatin inhibited the activation of microglia and astrocytes and reduced stroke injury (Piao et al., 2003) while also decreasing the expression of these inflammatory cytokines. Our results demonstrate that the pharmacological inhibition mediated by rosuvastatin attenuated HT, mainly via the NF-κB and MAPK pathways.

Our study does have some limitations. The possible neuroprotective mechanism of rosuvastatin in microglia and astrocytes, which might inhibit the inflammation mediated by MAPK phosphatase 1, salt-inducible kinase 2 (Ma et al., 2017) and low-density lipoprotein receptor-related protein 1 (Yepes et al., 2003; Cheng et al., 2006; Suzuki et al., 2009; Zhang et al., 2009), was not detected after ICH. In additional research, we will explore the potentially detailed molecular signaling pathway of rosuvastatin to further understand its potential clinical applications in HT therapy.

**Figure 9:**
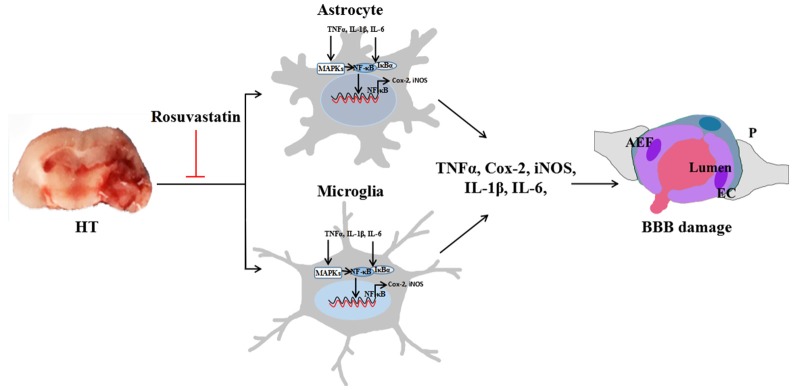
Rosuvastatin protected against HT via decreasing BBB leakage by attenuating inflammation. Astrocytic and microglial activation increase the release of inflammatory cytokines, such as TNF-α, Cox-2, iNOS, IL-1β, and IL-6. The NF-κB and MAPK pathways are involved in glial inflammation. Activated MAPKs can stimulate IκB kinase, release NF-κB dimers from the inactive cytoplasmic NF-κB/IκB complex and induce the nuclear translocation of NF-κB, thus decreasing BBB leakage. AEF indicates the end foot of the astrocyte, P indicates the pericyte, EC indicates the endothelial cell, and blood is shown flowing from the lumen, indicating BBB leakage.

In conclusion, the major findings of this study are that rosuvastatin, at both normal and high doses, contributes to preventing HT after intravenous rt-PA thrombolysis in mice with brain ischemia by reducing glial cell activation. Meanwhile, the high-dose rosuvastatin treatment showed better anti-inflammatory effects in HT than did the normal-dose rosuvastatin treatment. The beneficial effects are related to inhibition of the inflammation-related NF-κB and MAPK pathways (Figure 9). These results indicate that rosuvastatin should be considered a promising therapeutic agent for rescuing HT, but further studies are necessary to elucidate the target of rosuvastatin and expand its clinical applications.

## Author Contributions

DL and YL wrote the article. DL, YL, HM, JZ and LS performed the experiments. DL and YL contributed to sample collection and data analyses. YZ and AX designed the study and revised the manuscript. All authors approved the final version of the manuscript. We also thank American Journal Experts for editing and polishing of the manuscript (Submission No. RTTNDBG2).

## Conflict of Interest Statement

The authors declare that the research was conducted in the absence of any commercial or financial relationships that could be construed as a potential conflict of interest.
